# Medical adherence in the time of social distancing: a brief report on the impact of the COVID-19 pandemic on adherence to treatment in patients with diabetes

**DOI:** 10.20945/2359-3997000000362

**Published:** 2021-04-29

**Authors:** Debora Wilke Franco, Janine Alessi, Alice Scalzilli Becker, Bibiana Brino do Amaral, Giovana Berger de Oliveira, Beatriz D. Schaan, Gabriela Heiden Telo

**Affiliations:** 1 Pontifícia Universidade Católica do Rio Grande do Sul Porto Alegre RS Brasil Programa de Pós-graduação em Medicina e Ciências da Saúde, Pontifícia Universidade Católica do Rio Grande do Sul, Porto Alegre, RS, Brasil.; 2 Pontifícia Universidade Católica do Rio Grande do Sul Escola de Medicina Porto Alegre RS Brasil Escola de Medicina, Pontifícia Universidade Católica do Rio Grande do Sul, Porto Alegre, RS, Brasil.; 3 Universidade Federal do Rio Grande do Sul Porto Alegre RS Brasil Programa de Pós-Graduação em Ciências Médicas: Endocrinologia, Universidade Federal do Rio Grande do Sul, Porto Alegre, RS, Brasil.; 4 Universidade Federal do Rio Grande do Sul Escola de Medicina Porto Alegre RS Brasil Escola de Medicina, Universidade Federal do Rio Grande do Sul, Porto Alegre, RS, Brasil.; 5 Hospital de Clínicas de Porto Alegre Divisão de Endocrinologia Porto Alegre RS Brasil Divisão de Endocrinologia, Hospital de Clínicas de Porto Alegre, Porto Alegre, RS, Brasil.; 6 Pontifícia Universidade Católica do Rio Grande Hospital São Lucas Departamento de Medicina Interna Porto Alegre RS Brasil Departamento de Medicina Interna, Hospital São Lucas, Pontifícia Universidade Católica do Rio Grande, Porto Alegre, RS, Brasil.

**Keywords:** Medical adherence, COVID-19 pandemic, diabetes mellitus, adherence to treatment, social distancing

## Abstract

We conducted a cross-sectional study to evaluate the impact of social distancing determined by the COVID-19 pandemic on treatment adherence using the Self-Care Inventory-revised in adults with diabetes mellitus. In type 1 diabetes, the adherence score was lower during than before social distancing.

## INTRODUCTION

Treatment adherence is the main factor in the management of hyperglycemia and, consequently, in the reduction of diabetes-related complications. Stressful events have a significant impact on treatment adherence in patients living with diabetes mellitus, and the COVID-19 pandemic provides further reason for concern about these patients, who are among those at highest risk ( 1 - 5 ). In recent months, Brazil has become one of the pandemic epicenters in the world, with approximately 2,100,000 confirmed cases so far ( 6 ). This scenario may directly impact diabetes care due to poor availability of medical appointments, increased difficulty in obtaining medications, and home isolation.

The intrinsic differences in type of diabetes may impact patients with type 1 and type 2 diabetes differently during the pandemic. While spending more time at home could facilitate proper adherence to treatment guidelines, maintaining healthy eating habits and exercising may be challenging during quarantine. Therefore, the present study aimed to evaluate adherence in a cohort of patients with type 1 and type 2 diabetes during social distancing.

## METHODS

### Study design and setting

We conducted a controlled cross-sectional study to evaluate aspects of treatment adherence in a cohort of patients with type 1 and type 2 diabetes during social distancing. We invited patients to participate in the study by phone calls, during which the informed consent form was read aloud and any questions from potential participants were answered or clarified. Each patient's informed consent was documented through audio recording. We administered a specific questionnaire to evaluate treatment adherence 1 month after the publication of the national recommendation of social distancing for high-risk groups for COVID-19 in Brazil.

### Participants

We selected patients under regular monitoring at the endocrinology outpatient clinic of a tertiary public hospital in southern Brazil and divided them into 2 groups: social distancing group and control group. The inclusion criteria were previous diagnosis of type 1 or type 2 diabetes, age ≥18 years, HbA1c measured 3 months prior to inclusion in the study, and updated contact information in the electronic database. We excluded patients with any physical or cognitive impairment that could limit questionnaire administration and those who were hospitalized at the time of recruitment. For the social distancing group, we also excluded patients who were not following other social distancing rules besides the current national recommendation for high-risk groups. For the control group, we selected patients from 2 previous cohorts designed to evaluate treatment adherence before the pandemic. The data were collected in 2014 for the type 1 diabetes control group and in 2016 for the type 2 diabetes control group. Patients in the social distancing and control groups were matched for age for the present study.

### Study outcome

We used a validated Brazilian Portuguese version of the Self-Care Inventory-revised (SCI-R) ( 7 - 8 ) to evaluate treatment adherence before and during the pandemic. We asked participants to answer the 14 questions with frequency descriptors. A score of 1 to 5 is assigned to each item, and a final score ranging from 14 to 70 is then calculated. Higher scores indicate greater adherence to diabetes treatment.

### Clinical variables

We collected demographic and clinical variables from medical records and confirmed the data during the study telephone evaluation.

### Sample size

Considering an estimated 27% increase in the risk of non-adherence in situations of anxiety and depression and the prevalence of poor adherence described in the literature of 49.1% in type 1 diabetes and of 42.0% in type 2 diabetes, we calculated that 110 patients with type 1 diabetes and 150 patients with type 2 diabetes would be needed to perform an analysis with 80% power and alpha of 0.05 ( 9 - 11 ).

### Statistical analysis

We presented the data as mean (SD), median and interquartile range (IQR), or percentages. We used unpaired *t* test for continuous variables and χ^2^ test for categorical variables. We compared the social distancing and control groups using the Mann-Whitney test for nonparametric data. We stratified the analyses according to type of diabetes. Because the time of evaluation differed between the social distancing group and the control groups, we included a variable called ‘lendar year’ in a linear regression model to adjust for possible differences related to improvements in diabetes care over time. Considering that the effect of time is expected to be positive on treatment-adherence parameters, the adjustments for calendar year are presented only when the adherence score has increased in the social distancing group, as this group represents the most recently assessed one for diabetes care. We analyzed the data in SPSS, version 20, and set the level of statistical significance at p ≤ 0.05 for all analyses.

### Ethical aspects

The study was approved by the institution's research ethics committee (number 4.029.368) and reported following the STROBE guidelines ( 12 ). The project is registered at the Brazilian platform for research involving human participants called Plataforma Brasil ( https://plataformabrasil.saude.gov.br/login.jsf ), number 30528620.1.0000.5327.

## RESULTS

### Characteristics of the participants

We included 260 participants in the study. Overall, mean age was 43.7 (SD 12.7) years; 45.5% were female and 97.3% were white (see Table 1 ). The social distancing and control groups were similar in terms of demographics and clinical variables. There was also no difference in treatment regimens between the groups.

**Table 1:** Demographics and clinical characteristics of study participants

TYPE 1 DIABETES		Total (N = 110)	Social distancing group (n = 55)	Control (n = 55)	P value
Age (years)		43.7 ± 12.7	43.4 ± 13.8	43.9 ± 11.7	0.81
Sex (% female)		45.5%	49.1%	45.5%	0.44
Race/ethnicity (% white)		97.3%	96.4%	98.2%	0.22
Age at diabetes diagnosis (years)		19.2 ± 11.4	18.4 ± 12.5	19.9 ± 10.2	0.47
Diabetes duration (years)		24.6 ± 11.4	24.9 ± 11.8	24.1 ± 11.0	0.70
HbA1c (%)		8.6 ± 1.6	8.6 ± 1.5	8.6 ± 1.8	1.0
(mmol/mol)		71.0 ± 17.9	71.0 ± 15.9	71.0 ± 19.7	
Diabetes complications					
	Retinopathy	48.2%	49.1%	47.3%	0.83
	Neuropathy	17.3%	23.6%	10.9%	0.08
BMI overweigh/obese (%)		46.3%	45.3%	47.3%	0.83
Hypertension (%)		21.8%	25.5%	18.2%	0.35
Cardiovascular disease (%)		11.8%	12.7%	10.9%	0.76

TYPE 2 DIABETES		(N = 150)	(n = 75)	(n = 75)	
Age (years)		61.3 ± 7.9	61.8 ± 8.7	60.7 ± 7.0	0.40
Sex (% female)		60.7%	63.5%	58.7%	0.38
Race/ethnicity (% white)		74.0%	78.4%	70.7%	0.09
Age at diabetes diagnosis (years)		43.6 ± 10.6	42.8 ± 9.7	44.2 ± 11.4	0.42
Diabetes duration (years)		17.6 ± 9.0	18.8 ± 9.5	16.5 ± 8.6	0.12
HbA1c (%)		8.8 ± 1.5	8.9 ± 1.5	8.6 ± 1.5	0.36
(mmol/mol)		73.0 ± 16.4	74.0 ± 16.4	70.0 ± 16.4	
Diabetes complications					
	Retinopathy	46.0%	43.2%	48.0%	0.46
	Neuropathy	34.0%	29.7%	38.7%	0.39
	Nephropathy	46.7%	44.6%	49.3%	0.54
Insulin use (%)		84.7%	89.2%	80.0%	0.27
BMI overweigh/obese (%)		91.0%	91.3%	90.7%	0.94
Hypertension (%)		82.6%	82.4%	82.7%	0.89
Cardiovascular disease (%)		38.7%	41.9%	36.0%	0.55

Data are presented as mean ± SD or %. α ≤ 0.05 indicates significant difference. HbA1c: hemoglobin A1c; BMI: body mass index.

### Outcomes

**Figure 1:**
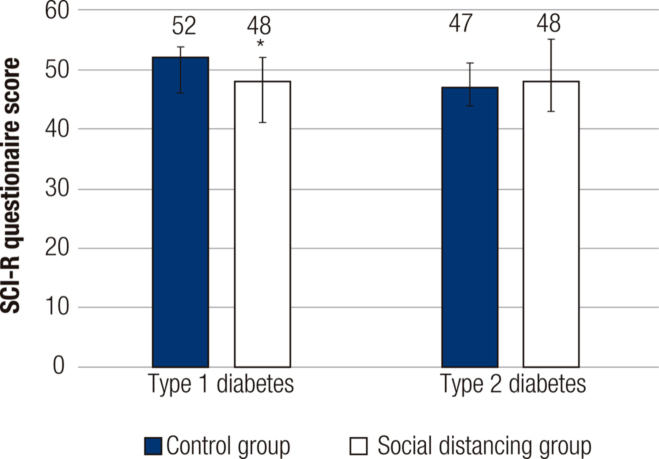
Adherence scores among patients with type 1 and type 2 diabetes before and during social distancing determined by COVID-19. Data are presented as median and interquartile range. *: α level ≥ 0.05, indicating significant difference. The validated Brazilian version of the Self-Care Inventory-revised (SCI-R) was used for this evaluation. Scores range from 14 to 70. Higher scores indicate greater adherence to diabetes treatment.

In type 1 diabetes, the median SCI-R score was lower in the social distancing group (48.0, IQR 41.0-52.0) than in the control group (52.0, IQR 46.0-54.0) (p < 0.01) (see Figure 1 ). In type 2 diabetes, the median SCI-R scores were similar in the social distancing (48.0, IQR 43.0-55.0) and control groups (47.0, IQR 44.0-51.0) (p = 0.14).

## DISCUSSION

This study found no difference in adherence scores among patients with type 2 diabetes before and during social distancing related to the COVID-19 pandemic. Although spending more time at home could facilitate proper adherence to treatment guidelines, adherence scores worsened among patients with type 1 diabetes during social distancing.

This study has some limitations. As the study has a cross-sectional design, our results reflect only associations between the pandemic and adherence to diabetes treatment, and not causal relationships. Also, the SCI-R was applied only 1 month after the start of social distancing. Considering that the effects of social distancing on adherence can be time dependent, it is possible that the scores would worsen over time. The SCI-R was originally validated for self-administration; therefore, its administration by telephone could introduce a potential measurement bias. Finally, the difference in the time of questionnaire administration between the social distancing and control groups may also have interfered with the results. Nevertheless, to the best of our knowledge, this is the first study to assess the impact of social distancing on treatment-adherence parameters in diabetes.

Reduced availability of multidisciplinary teams and increased difficulty in obtaining medical care during the pandemic may directly interfere with treatment adherence in the future. In addition, studies have shown an increase in psychological and eating disorders during quarantine, which may have an even greater long-term impact in patients with diabetes, especially by negatively impacting adherence to recommended diabetes self-care behaviors ( 13 - 14 ). Further studies are warranted to better understand the impact of home confinement on adherence parameters in diabetes.

## References

[1] Walders-Abramson N, Venditti EM, Levers-Landis CE, Anderson B, El Ghormli L, Geffner M (2014). Relationships among stressful life events and physiological markers, treatment adherence, and psychosocial functioning among youth with type 2 diabetes. J Pediatr.

[2] Pyatak EA, Sequeira PA, Whittemore R, Vigen CP, Peters AL, Weigensberg MJ (2014). Challenges contributing to disrupted transition from paediatric to adult diabetes care in young adults with Type 1 diabetes. Diabet Med.

[3] Fadini GP, Morieri ML, Longato E, Avogaro A (2020). Prevalence and impact of diabetes among people infected with SARS-CoV-2. J Endocrinol Invest.

[4] Singh A, Gupta R, Ghosh A, Misra A (2020). Diabetes in COVID-19: Prevalence, pathophysiology, prognosis and practical considerations. Diabetes Metab Syndr Clin Res Rev J.

[5] Muniyappa R, Gubbi S (2020). COVID-19 pandemic, coronaviruses, and diabetes mellitus. Am J Physiol Endocrinol Metab.

[6] World Health Organization Coronavirus disease (COVID-19) Situation Report-131.

[7] Teló GH, De Souza MS, Schaan BDA (2014). Cross-cultural adaptation and validation to Brazilian Portuguese of two measuring adherence instruments for patients with type 1 diabetes. Diabetol Metab Syndr.

[8] Teló GH, Iorra FQ, Velho BS, Sparrenberger K, Schaan BD (2020). Validation to Brazilian Portuguese of the self-care inventory-revised for adults with type 2 diabetes. Arch Endocrinol Metab.

[9] DiMatteo MR (2004). Variations in patients' adherence to medical recommendations: A quantitative review of 50 years of research. Med Care.

[10] Maoui A, Bouzid K, Abdelaziz A, Abdelaziz A (2019). Epidémiologie du Diabète de Type 2 au Grand Maghreb. Exemple de la Tunisie. Revue systématique de la littérature. J la Société Tunisienne des Sci Médicales.

[11] Almeda-Valdes P, Ríofrio JP, Coronado KWZ, la Parra DR de, Cabrera JB, Gómez-Pérez FJ (2019). Factors Associated with Insulin Nonadherence in Type 1 Diabetes Mellitus Patients in Mexico. Int J Diabetes Metab.

[12] Gharaibeh A, Koppikar S, Bonilla-Escobar FJ (2014). Strengthening the Reporting of Observational Studies in Epidemiology (STROBE) in the International Journal of Medical Students. Int J Med Students.

[13] Brooks SK, Webster RK, Smith LE, Woodland L, Wessely S, Greenberg N (2020). The psychological impact of quarantine and how to reduce it: rapid review of the evidence. Lancet.

[14] Vindegaard N, Benros ME (2020). COVID-19 pandemic and mental health consequences: Systematic review of the current evidence. Brain Behav Immun.

